# Clinical outcomes associated with failed thrombus aspiration during primary percutaneous coronary intervention

**DOI:** 10.1097/MD.0000000000046824

**Published:** 2026-01-02

**Authors:** Hasan Sohail, Kaleem Ullah Bacha, Syed Javaid Iqbal, Rashid Iqbal, Payal Bai, Dawood Umer, Abida Parveen, Jahanzeb Malik

**Affiliations:** a Department of Medicine, Ibn e Seena Hospital, Kabul, Afghanistan.

**Keywords:** cardiovascular outcomes, failed aspiration, primary PCI, STEMI, thrombus aspiration

## Abstract

Thrombus aspiration is performed during primary percutaneous coronary intervention (PCI) to improve outcomes in patients with ST-elevation myocardial infarction (STEMI). However, its success varies, and failed aspiration may impact prognosis. This study aimed to determine the predictors and clinical outcomes associated with failed thrombus aspiration. This retrospective study included 5323 patients who underwent primary PCI for STEMI. Patients were categorized into failed and successful thrombus aspiration groups. Baseline characteristics, angiographic findings, and procedural details were compared. Multivariate logistic regression identified independent predictors of aspiration failure, and clinical outcomes were analyzed over one-year. Failed thrombus aspiration occurred in 1176 patients (22.1%). Independent predictors included age ≥ 65 years, diabetes, hypertension, multi-vessel disease, high thrombus burden, and longer fluoroscopy time (all *P* < .001). Patients with failed aspiration had significantly higher in-hospital mortality (13.9% vs 5.6%), no-reflow (35.3% vs 6.5%), cardiogenic shock, and major adverse cardiovascular events (all *P* < .001). One-year mortality and heart failure hospitalizations were also increased. Failed thrombus aspiration is associated with worse short- and long-term outcomes in STEMI patients undergoing primary PCI. Identifying high-risk patients may help optimize procedural strategies and improve outcomes.

## 1. Introduction

Thrombus aspiration has been widely used during primary percutaneous coronary intervention (PCI) in patients with ST-segment elevation myocardial infarction (STEMI) with the aim of reducing thrombus burden, restoring optimal coronary blood flow, and minimizing distal embolization.^[1,2]^ Previous studies, including the TASTE and TOTAL trials, did not demonstrate a mortality benefit of routine aspiration.^[3,4]^ However, some evidence suggests that adjunctive aspiration may improve outcomes in selected patients with large thrombus burden.^[5]^ Despite this, the implications of *thrombus aspiration failure* – defined as the inability to retrieve visible thrombus or failure to improve thrombolysis in myocardial infarction (TIMI) flow – remain underrecognized.

Failed aspiration can leave behind substantial thrombus, leading to distal embolization, no-reflow, and impaired myocardial perfusion, all of which may adversely affect both short- and long-term outcomes.^[6,7]^ While the frequency and clinical significance of aspiration failure are poorly characterized, observational evidence suggests that high-risk patients – such as those with advanced age, diabetes, and multi-vessel disease – may be particularly vulnerable.^[8]^ Risk stratification tools and clinical scores, such as CHA_2_DS_2_-VASc, have shown predictive value for thrombotic complications in other cardiovascular conditions and may help identify patients at increased risk of aspiration failure.^[9,10]^ Similarly, whole blood viscosity has been associated with thrombus presence, further supporting the need for improved markers of thrombotic risk.^[11]^

This retrospective study therefore aims to evaluate the frequency, predictors, and clinical impact of thrombus aspiration failure during primary PCI for STEMI. By identifying adverse outcomes associated with aspiration failure, and highlighting potential risk stratification strategies, this work seeks to inform procedural decision-making and optimize treatment in this high-risk subgroup.

## 2. Methods

### 2.1. Study design and setting

This retrospective observational study was carried out at the Abbas Institute of Medical Sciences (Study ID # AIMS/24/80). The study population consisted of patients who underwent primary PCI for STEMI between January 2020 and August 2024. Ethical clearance was obtained from the institutional review board, and the study adhered to the ethical guidelines outlined in the Declaration of Helsinki. All participants provided written informed consent before inclusion in the study.

### 2.2. Patient population

Patients were included if they presented with STEMI and underwent thrombus aspiration during primary PCI. Characteristic electrocardiographic changes and elevated cardiac biomarkers confirmed STEMI diagnosis. Eligible patients were at least 18 years of age and had thrombus aspiration performed during PCI. Patients with non-NSTEMI or stable coronary artery disease, as well as those with incomplete medical records or inadequate follow-up data, were excluded. Additionally, patients who declined consent were not included.

### 2.3. Data collection

Data collection was conducted retrospectively from January 2020 to August 2024. The information was obtained from the hospital’s electronic medical record system and the catheterization laboratory database. Key data points included patient demographics, cardiovascular risk factors (such as diabetes, hypertension, and smoking status), and history of previous myocardial infarction or coronary revascularization. Detailed angiographic and procedural data were collected, including the location of the culprit coronary lesion, thrombus burden, and TIMI flow grades both before and after thrombus aspiration. The success or failure of thrombus aspiration was recorded, along with the use of adjunctive therapies, such as glycoprotein IIb/IIIa (GP IIb/IIIa) inhibitors and antiplatelet agents, and the need for stenting or other interventional strategies.

### 2.4. Outcome measures

The primary outcome was the frequency of thrombus aspiration failure, defined as either the inability to retrieve visible thrombus or the failure to improve TIMI flow post-aspiration. Secondary outcomes included short-term adverse events, such as in-hospital mortality, major adverse cardiovascular events (MACE), and the occurrence of the no-reflow phenomenon. Long-term outcomes, including mortality, reinfarction, and the development of heart failure, were also assessed over the follow-up period.

### 2.5. Statistical analysis

Statistical analyses were performed using standard methods to assess the impact of thrombus aspiration failure on both short-term and long-term outcomes. Continuous variables were reported as means and standard deviations, while categorical variables were presented as frequencies and percentages. Univariate and multivariate analyses were used to identify predictors of thrombus aspiration failure and adverse outcomes. Kaplan–Meier survival curves were employed to evaluate long-term event-free survival, with the log-rank test used to compare outcomes between groups. Statistical significance was defined as a *P*-value < .05.

## 3. Results

### 3.1. Baseline characteristics

A total of 5323 patients undergoing primary PCI for STEMI were included. The mean age was 60.1 ± 9.9 years, with 70.0% being male. The prevalence of cardiovascular risk factors was high, with 60.2% having hypertension, 71.8% diabetes mellitus, and 59.0% dyslipidemia. A history of prior myocardial infarction (MI) was reported in 79.7% of patients, and 41.1% had undergone a prior PCI (Table 1). The most common culprit artery was the left anterior descending artery (54.4%), and baseline TIMI flow grade 0 to 1 was observed in 84.6% of patients. Thrombus aspiration was attempted in all cases, with a failure rate of 22.1% (n = 1176).

**Table 1:** Baseline characteristics.

Characteristic	Overall (n = 5323)
Demographics	
Age (yr), mean ± SD	60.1 ± 9.9
Male, n (%)	3725 (70.0%)
Body mass index (BMI), mean ± SD	27.3 ± 4.5
Cardiovascular risk factors	
Hypertension, n (%)	3207 (60.2%)
Diabetes mellitus, n (%)	3823 (71.8%)
Smoking status, n (%)	
Current smoker	1876 (35.2%)
Former smoker	781 (14.7%)
Never smoker	2666 (50.1%)
Dyslipidemia, n (%)	3140 (59.0%)
Family history of CAD, n (%)	1568 (29.5%)
Chronic kidney disease, n (%)	932 (17.5%)
Prior stroke, n (%)	423 (7.9%)
Clinical presentation	
Systolic blood pressure (mm Hg), mean ± SD	129 ± 18
Diastolic blood pressure (mm Hg), mean ± SD	78 ± 12
Heart rate (bpm), mean ± SD	82 ± 15
Killip class at presentation, n (%)	
Class I	3930 (73.9%)
Class II	971 (18.2%)
Class III	283 (5.3%)
Class IV	139 (2.6%)
Laboratory findings	
Hemoglobin (g/dL), mean ± SD	13.8 ± 1.9
White blood cell count (×10^9^/L), mean ± SD	9.1 ± 3.2
Creatinine (mg/dL), mean ± SD	1.1 ± 0.4
LDL cholesterol (mg/dL), mean ± SD	118 ± 36
HbA1c (%), mean ± SD (in diabetics)	7.6 ± 1.8
Angiographic and procedural data	
Culprit artery, n (%)	
Left anterior descending (LAD)	2897 (54.4%)
Right coronary artery (RCA)	1594 (29.9%)
Left circumflex (LCx)	832 (15.7%)
Baseline TIMI flow 0–1, n (%)	4502 (84.6%)
Thrombus burden (grades 4–5), n (%)	3761 (70.7%)
Use of GP IIb/IIIa inhibitors, n (%)	1698 (31.9%)
Use of dual antiplatelet therapy, n (%)	5323 (100%)
Stent implantation, n (%)	4912 (92.3%)
No-reflow phenomenon, n (%)	683 (12.8%)
Prior cardiovascular history	
Prior myocardial infarction, n (%)	4243 (79.7%)
Prior PCI, n (%)	2189 (41.1%)
Prior CABG, n (%)	732 (13.8%)

CAD = coronary artery disease, CABG = coronary artery bypass grafting, GP IIb/IIIa = glycoprotein IIb/IIIa, HbA1c = hemoglobin A1c, LDL = low-density lipoprotein, PCI = percutaneous coronary intervention, TMI = thrombolysis in myocardial infarction.

### 3.2. Angiographic and procedural characteristics

Among the population, 53.8% had single-vessel disease, while 46.2% had multi-vessel disease. A high thrombus burden (grade 4–5) was observed in 70.7% of cases. Baseline TIMI 0 flow was present in 64.3% of cases. Post-PCI TIMI 3 flow was achieved in 76.5% of patients, whereas 8.6% had post-PCI TIMI 0 to 1 flow, indicating unsuccessful reperfusion. GP IIb/IIIa inhibitors were used in 31.9% of patients, and stent implantation was performed in 92.3%. The mean contrast volume used was 192 ± 55 mL, with an average fluoroscopy time of 18.5 ± 7.2 minutes (Table 2).

**Table 2:** Angiographic characteristics.

Angiographic Characteristic	Overall (n = 5323)
Culprit artery, n (%)	
Left anterior descending (LAD)	2897 (54.4%)
Right coronary artery (RCA)	1594 (29.9%)
Left circumflex (LCx)	832 (15.7%)
Number of diseased vessels, n (%)	
Single-vessel disease	2865 (53.8%)
Multi-vessel disease	2458 (46.2%)
Thrombus burden (angiographic grade), n (%)	
Grade 0–1 (minimal/none)	601 (11.3%)
Grade 2–3 (moderate)	961 (18.1%)
Grade 4–5 (large)	3761 (70.7%)
Baseline TIMI flow, n (%)	
TIMI 0 (complete occlusion)	3421 (64.3%)
TIMI 1 (minimal flow)	1081 (20.3%)
TIMI 2 (partial flow)	566 (10.6%)
TIMI 3 (normal flow)	255 (4.8%)
Post-PCI TIMI flow, n (%)	
TIMI 0–1	456 (8.6%)
TIMI 2	794 (14.9%)
TIMI 3	4073 (76.5%)
Thrombus aspiration attempted, n (%)	5323 (100%)
Failed thrombus aspiration, n (%)	1176 (22.1%)
Use of GP IIb/IIIa inhibitors, n (%)	1698 (31.9%)
Stent implantation, n (%)	4912 (92.3%)
Direct stenting, n (%)	1324 (24.9%)
Bailout stenting, n (%)	725 (13.6%)
Pre-dilation performed, n (%)	2439 (45.8%)
Post-dilation performed, n (%)	1897 (35.6%)
No-reflow phenomenon, n (%)	683 (12.8%)
Contrast volume (mL), mean ± SD	192 ± 55
Fluoroscopy time (min), mean ± SD	18.5 ± 7.2

GP IIb/IIIa = glycoprotein IIb/IIIa, PCI = percutaneous coronary intervention, TMI = thrombolysis in myocardial infarction.

### 3.3. Predictors of thrombus aspiration failure

Patients with aspiration failure were more likely to be older (≥65 years: 46.1% vs 30.3%, odds ratio (OR) = 1.89, 95% confidence interval (CI): 1.65–2.18, *P* < .001) and male (79.3% vs 73.7%, OR = 1.32, 95% CI: 1.14–1.52, *P* < .001). Diabetes mellitus (48.2% vs 33.6%, OR = 1.85, 95% CI: 1.61–2.12, *P* < .001), hypertension (61.7% vs 49.4%, OR = 1.63, 95% CI: 1.42–1.88, *P* < .001), and multi-vessel disease (62.5% vs 41.6%, OR = 2.35, 95% CI: 2.04–2.72, *P* < .001) were significant predictors. High thrombus burden (grade 4–5) was strongly associated with failure (88.8% vs 65.5%, OR = 4.1, 95% CI: 3.40–4.95, *P* < .001). The no-reflow phenomenon was significantly higher in the failure group (35.3% vs 6.5%, OR = 7.93, 95% CI: 6.65–9.45, *P* < .001) (Table 3).

**Table 3:** Predictors of failed thrombus aspiration.

Predictor variable	Failed thrombus aspiration (n = 1176, 22.1%)	Successful thrombus aspiration (n = 4147, 77.9%)	Odds ratio (OR)	95% CI	*P*-value
Age ≥ 65 yr, n (%)	542 (46.1%)	1257 (30.3%)	1.89	1.65–2.18	<.001
Male sex, n (%)	933 (79.3%)	3058 (73.7%)	1.32	1.14–1.52	<.001
Diabetes mellitus, n (%)	567 (48.2%)	1395 (33.6%)	1.85	1.61–2.12	<.001
Hypertension, n (%)	726 (61.7%)	2048 (49.4%)	1.63	1.42–1.88	<.001
Current smoking, n (%)	497 (42.3%)	1722 (41.5%)	1.03	0.90–1.18	.69
Previous MI, n (%)	328 (27.9%)	902 (21.8%)	1.38	1.17–1.62	<.001
Multi-vessel disease, n (%)	735 (62.5%)	1723 (41.6%)	2.35	2.04–2.72	<.001
Culprit artery – LAD, n (%)	694 (59.0%)	2203 (53.1%)	1.26	1.10–1.45	.001
Baseline TIMI 0–1 flow, n (%)	988 (84.0%)	3514 (84.7%)	0.95	0.80–1.12	.54
Thrombus burden grade 4–5, n (%)	1045 (88.8%)	2716 (65.5%)	4.1	3.40–4.95	<.001
No-reflow phenomenon, n (%)	415 (35.3%)	268 (6.5%)	7.93	6.65–9.45	<.001
Use of GP IIb/IIIa inhibitors, n (%)	567 (48.2%)	1131 (27.3%)	2.46	2.14–2.83	<.001
Fluoroscopy time (min), mean ± SD	21.4 ± 8.1	17.3 ± 6.4	1.07	1.05–1.09 (per min)	<.001
Contrast volume (mL), mean ± SD	214 ± 60	185 ± 52	1.02	1.01–1.03 (per 10 mL)	<.001

CI = confidence interval, GP IIb/IIIa = glycoprotein IIb/IIIa, LAD = left anterior descending, MI = myocardial infarction, TMI = thrombolysis in myocardial infarction.

### 3.4. Clinical outcomes

Patients with aspiration failure had significantly worse in-hospital and long-term outcomes. In-hospital mortality was higher (13.9% vs 5.6%, OR = 2.74, 95% CI: 2.22–3.38, *P* < .001), along with increased MACE (26.8% vs 17.3%, OR = 1.75, 95% CI: 1.50–2.05, *P* < .001). The no-reflow phenomenon was markedly more frequent in the failure group (35.3% vs 6.5%, OR = 7.93, 95% CI: 6.65–9.45, *P* < .001). Cardiogenic shock (16.1% vs 8.5%, OR = 2.06, 95% CI: 1.70–2.49, *P* < .001) and acute heart failure (21.6% vs 12.4%, OR = 1.94, 95% CI: 1.64–2.28, *P* < .001) were also significantly more common.

At one year, long-term mortality was higher in the failure group (23.1% vs 14.2%, OR = 1.82, 95% CI: 1.55–2.14, *P* < .001). Heart failure hospitalization (25.6% vs 15.6%, OR = 1.84, 95% CI: 1.56–2.17, *P* < .001) and target vessel revascularization (18.2% vs 12.8%, OR = 1.51, 95% CI: 1.27–1.79, *P* < .001) were also higher. Reinfarction within 30 days was more frequent in the failure group (11.2% vs 6.5%, OR = 1.82, 95% CI: 1.45–2.29, *P* < .001) (Table 4).

**Table 4:** Outcomes.

Outcome	Failed thrombus aspiration (n = 1176, 22.1%)	Successful thrombus aspiration (n = 4147, 77.9%)	Odds ratio (OR)	95% CI	*P*-value
In-hospital mortality, n (%)	164 (13.9%)	234 (5.6%)	2.74	2.22–3.38	<.001
Major adverse cardiovascular events (MACE), n (%)	315 (26.8%)	718 (17.3%)	1.75	1.50–2.05	<.001
No-reflow phenomenon, n (%)	415 (35.3%)	268 (6.5%)	7.93	6.65–9.45	<.001
Stroke, n (%)	46 (3.9%)	77 (1.9%)	2.1	1.44–3.07	<.001
Cardiogenic shock, n (%)	189 (16.1%)	351 (8.5%)	2.06	1.70–2.49	<.001
Acute heart failure, n (%)	254 (21.6%)	516 (12.4%)	1.94	1.64–2.28	<.001
Reinfarction (30 d), n (%)	132 (11.2%)	268 (6.5%)	1.82	1.45–2.29	<.001
Target vessel revascularization (1 yr), n (%)	214 (18.2%)	529 (12.8%)	1.51	1.27–1.79	<.001
Long-term mortality (1 yr), n (%)	272 (23.1%)	589 (14.2%)	1.82	1.55–2.14	<.001
Heart failure hospitalization (1 yr), n (%)	301 (25.6%)	645 (15.6%)	1.84	1.56–2.17	<.001

CI = confidence interval.

## 4. Discussion

The present study comprehensively analyzes the frequency, predictors, and outcomes of thrombus aspiration failure in patients undergoing primary PCI for STEMI.^[10]^ Thrombus aspiration failure was observed in approximately 22.1% of cases and was associated with worse angiographic and clinical outcomes. Independent predictors included advanced age, diabetes mellitus, multi-vessel disease, high thrombus burden, and prolonged fluoroscopy time.^[11]^ Patients experiencing aspiration failure demonstrated significantly higher rates of in-hospital mortality, no-reflow phenomenon, cardiogenic shock, and MACE.^[12]^ These associations underscore the potential role of ineffective thrombus removal in worsening microvascular perfusion and prognosis, although causality cannot be firmly established.

The strong relationship between aspiration failure and the no-reflow phenomenon suggests that residual thrombotic material may contribute to distal embolization and impaired myocardial perfusion despite epicardial vessel recanalization.^[13]^ Similarly, the link between high thrombus burden and aspiration failure emphasizes the clinical challenge of managing these high-risk lesions.^[14]^ However, it remains uncertain whether aspiration failure is a direct cause of adverse outcomes or a marker of more complex disease and higher baseline risk.

Our results are broadly consistent with prior reports, with failure rates aligning with published data despite some variability related to operator expertise, device selection, and definitions of thrombus burden.^[15]^ Importantly, large randomized trials such as TASTE and TOTAL did not demonstrate mortality benefit with routine aspiration.^[3,4]^ These findings suggest that thrombus aspiration itself may not be universally beneficial, but our study highlights that when aspiration fails, patients appear particularly vulnerable to adverse events.^[16]^ This distinction underscores the need to refine patient selection and procedural strategies rather than abandoning thrombus aspiration entirely. Of note, some observational data suggest that selected patients with large thrombus burden may still derive benefit from adjunctive aspiration strategies.^[17]^

The higher incidence of rescue strategies following aspiration failure likely reflects attempts to mitigate suboptimal results; however, whether such interventions alter outcomes remains unclear.^[18]^ Furthermore, the association between aspiration failure and poor long-term outcomes, including reinfarction, heart failure hospitalization, and mortality at one year, suggests that its impact extends beyond the acute phase.^[19]^ Persistent microvascular dysfunction and residual thrombus may explain these observations, though further research is warranted to establish causality.

Future studies should explore whether alternative reperfusion approaches – such as advanced thrombectomy devices, adjunctive pharmacotherapies, or personalized procedural strategies – can improve outcomes in patients at high risk for aspiration failure.^[20]^ Incorporating risk stratification tools may also help identify vulnerable patients before intervention. For example, the CHA_2_DS_2_-VASc score, originally developed for atrial fibrillation, has shown predictive value in mechanical mitral valve thrombosis,^[16]^ and whole blood viscosity has been associated with thrombus presence in echocardiographic studies.^[21]^ Integrating such risk scores and biomarkers with angiographic predictors may refine patient selection and inform tailored interventional strategies.

### 4.1. Limitations

This study has several limitations. First, its retrospective design may introduce selection bias, as procedural and clinical decisions were made at the discretion of the operators rather than being standardized. Second, the study was conducted at a single center, which may limit the generalizability of the findings to other populations with different operator experience and institutional protocols. Third, the definition of thrombus aspiration failure was based on angiographic assessment, which may not fully capture the microvascular consequences of aspiration failure. Fourth, the use of adjunctive pharmacologic therapies, including GP IIb/IIIa inhibitors, was not randomized, making it difficult to determine their true impact on outcomes in patients with aspiration failure.

In addition, operator technique, device selection, and variability in aspiration catheter performance were not systematically assessed. These factors are likely to have influenced the likelihood of aspiration success or failure and may represent important procedural confounders. Furthermore, the possibility of unmeasured clinical variables – such as thrombus composition, timing of presentation, and underlying microvascular function – cannot be excluded and may have affected both procedural outcomes and long-term prognosis. Finally, long-term follow-up data were limited to one year, and further studies with extended follow-up are needed to assess the durability of adverse outcomes in this patient population.

## 5. Conclusion

Thrombus aspiration failure during primary PCI for STEMI was associated with significantly worse short- and long-term outcomes, including higher mortality, increased rates of no-reflow, and MACE. Patients with large thrombus burden, multi-vessel disease, and hemodynamic instability were at higher risk of aspiration failure. The findings highlight the need for improved strategies to manage high-risk thrombotic lesions. Further research is required to refine thrombus management techniques and optimize patient outcomes. A tailored approach incorporating novel adjunctive therapies and risk stratification tools may help mitigate the adverse effects of aspiration failure.

## Author contributions

**Conceptualization:** Kaleem Ullah Bacha, Payal Bai, Dawood Umer, Abida Parveen, Jahanzeb Malik.

**Investigation:** Abida Parveen.

**Resources:** Payal Bai.

**Supervision:** Rashid Iqbal.

**Validation:** Syed Javaid Iqbal.

**Visualization:** Hasan Sohail, Syed Javaid Iqbal, Abida Parveen.

**Writing** – **review & editing:** Hasan Sohail, Kaleem Ullah Bacha, Syed Javaid Iqbal, Rashid Iqbal, Payal Bai, Dawood Umer, Abida Parveen, Jahanzeb Malik.
